# Recent Advances in p53

**DOI:** 10.3390/biom11020211

**Published:** 2021-02-03

**Authors:** Gabriella D’Orazi

**Affiliations:** 1 Department of Research, Unit of Cellular Networks, IRCCS Regina Elena National Cancer Institute, 00144 Rome, Italy; gdorazi@unich.it; 2 Department of Neurosciences, Imaging and Clinical Sciences, University G. D’Annunzio, 00131 Chieti, Italy

Tumor suppressor protein p53 (TP53) is a key transcription factor that, in response to various stress signals, regulates numerous genes involved in a broad range of cellular functions including DNA repair, apoptosis, cell cycle arrest, senescence, metabolism, etc. Since the presence of a functional p53 pathway is incompatible with tumor growth, the *TP53* gene is often mutated in cancers mostly in the DNA binding domain (DBD), to avoid binding to canonical target gene promoters [1]. Some hotspot p53 mutants (mutp53) (i.e., R273H) may still regulate gene transcription, although not in a direct way, rather by interacting with other transcription factors, such as c-Myc, NF-Y, NRF2, STAT3, etc.; such interaction promotes the transcription of genes involved in cell growth, resistance to apoptosis and metabolic reprogramming [2]. Therefore, some p53 mutant proteins may not only lose the p53 oncosuppressor wild-type (wtp53) activity but also acquire oncogenic functions that promote cancer progression and resistance to therapies [3]. In this regard, understanding the many ways of inactivation of p53 oncosuppressor functions has become, in the last years, a challenging field of research in the attempt to find drugs/small molecules able to inactivate mutp53 or reactivate the p53 wild-type function and; therefore, pave the way for clinical application in cancer patients.

This Special Issue includes six original research papers and seven review articles from well-known experts in the field providing the reader with advances in understanding p53 function and dysfunction, for clinical translational purposes and perspectives for future research.

*TP53* mutations are associated with poor prognosis in 75% of the pancreatic ductal adenocarcinoma (PDAC) patients, and the study by Butera et al. [4] explored the functional effect of the hot-spot p53 mutant isoforms R175H and R273H on cancer cell secretome. By comparing the secretome of p53-null PDAC cells after ectopic overexpression of R175H-mutp53 or R273H-mutp53, using high-resolution SWATH-MS technology, the study shows several differentially secreted proteins, among which 15 are common to both mutants, reported to promote cancer progression and epithelial–mesenchymal transition.; therefore, the role of mutp53 in regulating the cancer–stroma relationship through a specific secretome that promotes cancer aggressiveness is underlined. That secretome might become a promising biomarker secreted signature in PDAC patients, predictive of p53 mutations that hopefully could be inactivated by specific target therapies.

The p53 secretome topic is illustrated in an in-depth review [5], showing that many mutant p53 proteins shape a tumor cell secretome that creates a supportive microenvironment at the primary tumor site and primes niches in distant organs for future metastatic colonization. Mutp53, by controlling the secretion of soluble and vesicle-bound proteins, has widespread non-cell-autonomous effects on the cellular microenvironment, ranging from remodeling of the extracellular matrix (ECM) to cell–cell communication. On the other hand, antiproliferative target genes of p53 orchestrate the senescence program, which not only permanently arrests cell proliferation but also triggers secretion of proteins, collectively defined as the senescence-associated secretory phenotype (SASP), that transform the microenvironment in a non-cell-autonomous manner, limiting tumorigenesis. As such, mutp53 and mutp53-triggered cell-extrinsic pathways emerge as even more interesting targets for the treatment of highly aggressive cancer types.

Senescence is a permanent cell-cycle arrest that has a crucial role in aging, and it also represents a robust physiological antitumor response, which counteracts oncogenic insults. The review by Mijit et al. [6] highlights the crucial role of senescence and of p53-induced senescence in physiological and pathological processes. The review also underlines that senescent cells, although permanently arrested, are still metabolically active and secrete a variety of pro-inflammatory or proteolytic molecules to communicate with the tissue microenvironment and the neighboring cells, ultimately triggering tissue dysfunction and/or unfavorable outcomes. Additionally, in this study, the promising role of secretome in the cell–microenvironment interaction, to address different and more effective therapeutic strategies in aging related disorders, is underlined.

In the attempt to define whether *TP53* mutations are suitable as biomarkers for another type of cancer, that is, the high-grade serous ovarian carcinomas (HGSOC), the most lethal malignancy among gynecological cancers in the Western world, the study by Vitale et al. [7] aimed at identifying an optimal workflow to detect *TP53* mutations in baseline and longitudinal serum cell free DNA (cfDNA). This was conducted by analyzing tissues and archived sera from HGSOC patients using a next-generation sequencing (NGS) workflow alone or in combination with digital PCR (dPCR). The study reports that *TP53* mutations were present at diagnosis but became undetectable in cfDNA after chemotherapy and re-appeared at disease progression. The study, although conducted with a small sample of tissues, highlights the potential role of *TP53* missense mutations as a biomarker for clinical disease monitoring in ovarian cancer, and suggests that cfDNA has the potential to be a highly specific early molecular response marker in HGSOC, worthy to be studied further.

MicroRNAs (miRs) are short non-coding RNAs involved in gene expression, controlling many processes at the basis of the cell cycle, such as differentiation, development, and apoptosis [8]. Some miRs have been found to be involved in the network of the tumor suppressor p53. The study by Bizzarri et al. [9] attempted to investigate the interactions between the p53 DBD and miR4749, by fluorescence spectroscopy combined with computational modeling and docking. Through Förster resonance energy transfer (FRET) and a successive docking refinement, the authors put into evidence the formation of a specific complex between DBD and miR4749. They hypothesize that the interaction of miR4749 with p53 could impair the p53 DNA binding function leading to inhibition of the p53 oncosuppressor function. The direct action of miR4749 on p53 might inspire new therapeutic strategies finalized to restore the p53 anticancer function.

The study by Farooqi et al. [10] attempted to investigate if a yeast protein would show p53 DNA binding homology. The authors present the first evidence that a protein in *Saccharomyces cerevisiae* has increased capability after DNA damage of recognizing and binding specifically to known p53 binding sites. The clinical importance of this finding is that if the ancient DNA damage response can be seen to contain a protein with functional, but not DNA sequence, homology to p53, then this can be used to understand important functional properties of this critical tumor suppressor pathway.

An important field of study is the targeting of mutp53 to induce its degradation or to reestablish the wild-type function. The review by Loh [11] summarizes the mechanisms by which missense mutations inactivate p53, focusing on those targeting mutant DBD with the intended effect of restoring WT-like conformation and function. The stability class and the zinc-binding class of p53 mutants are analyzed, underscoring the need to classify p53 mutants based on functional and biophysical data as well as on structural location. Changing the conformation of the zinc binding site, through for instance zinc metallochaperone-1 (ZMC1) molecules, was shown to reactivate mutp53, while rescuing DNA-contact mutants has been more challenging.

Changing the conformation of the zinc binding site through, for instance, zinc metallochaperone-1 (ZMC1), which was shown to reactivate mutp53 while rescuing DNA-contact mutants, has been more challenging. However, more studies are necessary to classify mutp53 proteins in order to find those that will best respond to class-specific molecules and help stratify patients for personalized medicine.

Parallel to the Loh review, the review by Silva et al. [12] attempted to summarize the currently adopted methods for the activation and reactivation of the p53 tumor suppressor function, focusing on the synthetic approaches to obtain the small molecules used as reactivators. Since mutp53 can undergo aggregation similarly to that observed with other amyloid proteins, compounds and peptides that inhibit mutp53 aggregation are also described. Thus, the misfolded and aggregated states of mutp53 have become highly promising targets for the development of novel therapeutic strategies against cancer. The molecules tested so far have not yet reached the market and the effects can still be improved in terms of lower toxicity to normal cells, potency and affinity to targets. From the same group, it is addressed in another review how misfolding and prion-like amyloid aggregation of p53 seem to play a crucial role in cancer development [13]. Thus, proposing that the development of direct aggregation inhibitors may be aided by determination of the physiological conditions that trigger p53 amyloid conversion, and the atomistic structures of the pathological species.

Examples of mutp53 reactivation are then shown in the Garufi et al. study [14], where it is reported that a zinc–curcumin compound is able to induce mutp53H373 degradation through a mechanisms involving endoplasmic reticulum (ER) stress and autophagy, highlighting their interplay. As the activation of the ER stress/unfolded protein response (UPR) has both pro-survival and pro-death effects, caution is necessary in the design of therapies that target UPR components to increase the antitumor response, as they could hamper mutp53 degradation. Degradation of mutp53 is also shown in the study by Romeo et al. [15]. The authors find that phenylbutyrate (PBA), a derivative of butyric acid (BA), induces mutp53 degradation that correlates with cell death in glioblastoma cells. Therefore, suggesting the use of PBA in cancer patients, since this drug, already approved by FDA for the treatment of urea cycle disorders, is able to penetrate the brain–blood barrier [15].

The review by Di Agostino [16] highlights how mutp53 is involved in the expression of specific long non-coding RNAs (lncRNAs) to gain oncogenic functions through the creation of a complex network of pathways that influence each other. Therefore, applying combined treatments which target both the mutp53 proteins and the pathways modulated by the non-coding RNAs might be a novel anticancer strategy.

Finally, in the review by Cordani et al. [17], the effect of mutp53 on reactive oxygen species (ROS) and how ROS enhancement driven by mutp53 might represent an “Achilles heel” of cancer cells is summarized, suggesting the use of pro-oxidant drugs as a therapeutic approach for cancer patients bearing the mutant TP53 gene.

Altogether, the studies included in this Special Issue illustrate examples of the recent progress on the effect of p53 deregulation and the mechanisms underlining. They also summarize molecular mechanisms of p53 technological advances and applications of different approaches to obtain novel drugs for clinical purpose.
